# New advances in asymmetric organocatalysis II

**DOI:** 10.3762/bjoc.21.60

**Published:** 2025-04-15

**Authors:** Radovan Šebesta

**Affiliations:** 1 Department of Organic Chemistry, Faculty of Natural Sciences, Comenius University in Bratislava, Mlynská dolina, Ilkovičova 6, 842 15 Bratislava, Slovakia

**Keywords:** asymmetric organocatalysis, covalent activation, noncovalent activation

Organocatalysis is commonly defined as a form of catalysis where a small organic molecule, an organocatalyst, accelerates a chemical reaction. Unlike previously regarded traditional catalysts involving metals or enzymes, organocatalysts are composed of nonmetal elements, such as carbon, hydrogen, nitrogen, oxygen, phosphorus, or sulfur. The year 2000 is typically regarded as the birth of organocatalysis, which was at that time regarded as a new mode of action for chemical catalysts. In that year, List and MacMillan et al. published their landmark studies on proline- and imidazolidine-catalyzed aldol, Mannich, and cycloaddition reactions [1–4]. The history of scientific discoveries, however, is rarely defined by single specific dates that can unambiguously be connected to the discoveries. Instead, the chronology often seems to be quite convoluted but also ultimately more interesting, as can also be seen for organocatalysis. Even toward the end of the 20th century, there have been a few pioneering studies that should be counted as examples of asymmetric organocatalysis. The works of Jacobsen, Miller, Shi, and Denmark et al. marked the early sparks of interest in this type of chemistry based on catalysis by peptides, carbohydrates, or thiourea [5–8]. Going even more back in time, we shall recognize the work of Eder, Sauer, Wiechert, Hajos, and Parrish on the proline-catalyzed Robinson annulation in the 1970s [9–10]. In light of this, organocatalysis appears to be 50 rather than 25 years old. But is even that correct? Or should we look even more deeply into its history? Well, we can go even further back in time and acknowledge the inspirations from Kuhn and Langebeck in the 1930s, which ultimately leads us to research by Knoevenagel published in 1896 [11–12]. Another interesting historical note is tied to the now prominent Hayashi–Jørgensen catalyst. This prolinol silyl ether was independently discovered by the respective Hayashi and Jørgensen research teams in 2005 [13–14]. Interestingly, the use of prolinol alkyl ethers for asymmetric Michael additions, although at the time added in a stoichiometric manner, has already been described by Seebach et al. in 1982 [15]. From this perspective, organocatalysis is more than 100 years old, building on a long thread of illustrious past discoveries.

Asymmetric organocatalysis is now considered one of the three main pillars of asymmetric catalysis, along with metal-catalyzed reactions and biocatalysis. In the past 25 years, organocatalysis has grown rapidly into a broad area of research, with industrial applications now being developed [16]. The usefulness of any idea or methodology is ultimately often measured by its applications. The robustness, reliability, as well as the eco- and user-friendliness of asymmetric organocatalysis contribute to its growing applicability in the synthesis of complex target molecules, such as therapeutic agents [17–18]. However, nowadays organocatalysis does go well beyond the boundaries of organic chemistry. This can also be seen from a brief Web of Science analysis of the 6,700 articles, published within the past 25 years, retrieved through a search of the keywords “asymmetric organocatalysis”. Organocatalysis is now part of various areas of chemistry, spanning as far as polymer, materials, as well as green and sustainable science and technology (Figure 1).

**Figure 1:**
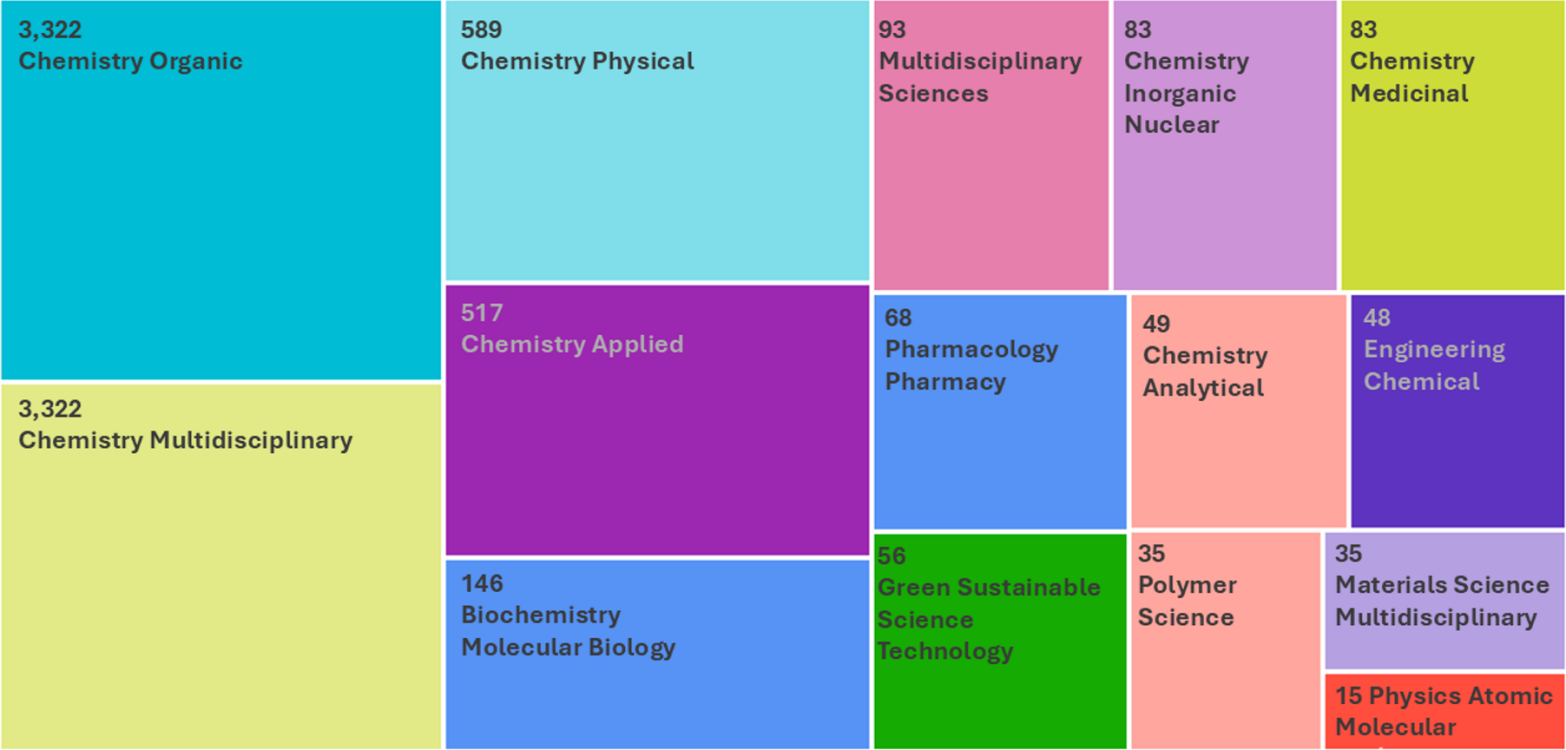
TreeMap chart of the top 15 Web of Science categories for 6,700 articles containing the keywords “asymmetric organocatalysis” published in 2001–2025.

In 2012, there was a thematic issue in the *Beilstein Journal of Organic Chemistry* devoted to asymmetric organocatalysis edited by one of the pioneers in this field, Benjamin List [19]. After a decade, another thematic issue was devoted to new advances in this field [20]. In 2022, three Review articles and nine research papers showcased the diversity and breadth into which asymmetric organocatalysis had grown. This follow-up thematic issue “New advances in asymmetric organocatalysis II” aims to monitor the progress in this exciting area and highlights excellent contributions in stereoselective organocatalytic transformations. The collection contains nine articles featuring various aspects of asymmetric organocatalysis.

In the first contribution, Waser et al. examined how chiral phase-transfer catalysts promote β-selective additions of azlactones to allenoates. Maruoka´s quaternary ammonium salts provided the corresponding substituted azlactones comprising a quaternary stereogenic center with the highest enantiomeric purity [21]. A contribution by Chowdhury and Dubey further underscored the importance of heterocyclic moieties in chiral compounds. Their article describes the enantioselective Michael addition of pyrazoline-5-ones to α,β-unsaturated ketones. The enantioselectivity and chemical efficiency of this transformation were achieved with a cinchona-alkaloid-derived primary-amine–Brønsted acid composite [22].

A good demonstration of how organocatalysis progressed from the original amine catalysts is the work of Shirakawa and co-workers. In this contribution, the authors employed a binaphthalene-derived sulfide organocatalyst for enantioselective bromolactonizations of α- and β-substituted 5-hexenoic acids to produce the corresponding chiral lactones [23]. Kowalczyk and co-workers showed how asymmetric organocatalysis can benefit from mechanochemical activation. They established that Michael additions of thiomalonates to enones, catalyzed by cinchona-derived primary amines, is efficient and enantioselective under ball-milling conditions [24]. Kondratyev and Malkov reviewed the recent progress in the organocatalytic synthesis of chiral homoallylic amines. This important structural motif is typically made by asymmetric allylation of imines, and the authors describe various catalytic approaches as well as applications of these strategies in total synthesis [25]. The enantioselective addition of propargyltrichlorosilane to aldehydes was studied by Prabhakar, Takenaka, and co-workers. This transformation was catalyzed by a biisoquinoline *N*,*N*’-dioxide catalyst, which acted as a chiral Lewis base [26]. Torres-Oya and Zurro reviewed the recent developments in organocatalytic cycloaddition reactions of unsaturated imines. A broad variety of activation modes, as well as catalyst structures, was covered and found to be useful in affording a diverse array of chiral N-heterocycles [27]. In my group, we recently became interested in atroposelective catalytic syntheses. Therefore, my team reviewed the recent progress in organocatalytic syntheses of axially chiral compounds [28]. The final article within this thematic issue was contributed by Tsogoeva, Harder, and co-workers. They developed and mechanistically studied the hydrocyanation of hydrazones. This interesting transformation was catalyzed by a calcium complex of BINOL phosphate [29].

As can be seen from the summary above, the articles in this thematic issue cover a diverse array of topics in contemporary asymmetric organocatalysis. Moreover, they also reflect the diversity of our scientific community, as researchers at various career stages are involved. Contributions to this issue also highlight its truly global character, with research teams from nine countries (Austria, India, Japan, Poland, UK, USA, Spain, Slovakia, and Germany) across three continents contributing to this remarkable selection.

As guest editor of this thematic issue, I am grateful to all authors for their excellent contributions. I sincerely thank the referees for providing their expertise and time, and the team at the *Beilstein Journal of Organic Chemistry* for their outstanding professionalism and support.

Radovan Šebesta

Bratislava, March 2025

## Data Availability

Data sharing is not applicable as no new data was generated or analyzed in this study.
